# Relationship between nausea and vomiting and physical activity in patients with lung cancer undergoing first chemotherapy

**DOI:** 10.3389/fonc.2024.1396637

**Published:** 2024-07-24

**Authors:** Liandi Bai, Li Ni, JianHong Lu, Yue Yu Zhang, Yuanyuan Yin, WeiYing Zhang, Xia Duan

**Affiliations:** ^1^ Department of Thoracic Surgery, Shanghai East Hospital, School of Medicine, Tongji University, Shanghai, China; ^2^ Department of nursing, Shanghai East Hospital, Tongji University School of Medicine, Shanghai, China; ^3^ Shanghai Key Laboratory of Maternal Fetal Medicine, Shanghai Institute of Maternal-Fetal Medicine and Gynecologic Oncology, Shanghai First Maternity and Infant Hospital, School of Medicine, Tongji University, Shanghai, China

**Keywords:** lung cancer, nausea, vomiting, platinum, physical activity

## Abstract

**Objective:**

Nausea and vomiting are the distressing and debilitating side effects of chemotherapy. This study explores the relationship between the degree of nausea and vomiting and physical activity in patients with lung cancer during the first chemotherapy cycle.

**Design:**

A total of 107 patients with lung cancer who received platinum drugs during chemotherapy in a hospital in Shanghai, China, in 2023 were involved in this study. Data were collected with medical record system and self-reported questionnaires.Questionnaires included the International Physical Activity Questionnaire (IPAQ) and Index of Nausea, Vomiting, and Retching (INVR). IPAQ was used before chemotherapy; INVR was used on the second and sixth day of chemotherapy, followed by the analysis of the correlation between physical activity status and degree of nausea and vomiting during chemotherapy. The influencing factors of nausea and vomiting during chemotherapy in patients with lung cancer were analyzed using logistic regression analysis.

**Results:**

More than half of the patients experienced nausea, vomiting or retching related symptoms after chemotherapy, and the proportion of moderate to high physical activity was 50.5%. Univariate analysis showed that the degree of nausea and vomiting was influenced by factors such as age, gender, and history of drinking (P < 0.05). The degree of nausea and vomiting was negatively correlated with physical activity (P < 0.05). The multivariate linear analysis showed that gender, history of drinking, and moderate and high physical activity are contributing factors to nausea and vomiting during chemotherapy (P < 0.05).

**Conclusion:**

Moderate and high physical activity before chemotherapy is a protective factor for nausea and vomiting in patients with lung cancer. Physical activity can not only improve the degree of nausea and vomiting in patients with lung cancer but also reduce the incidence in these patients during the first chemotherapy cycle.

## 1 Introduction

Lung cancer is a malignant tumor accounting for the highest morbidity and mortality worldwide (1). According to the latest cancer data, approximately 820,000 new cases of lung cancer and 710,000 deaths were observed in 2020, thereby ranking first in the number of new cases and deaths by malignant tumors (1). Among all the new cases of malignant tumors, lung cancer ranked first, accounting for 18.06%, and the total number of malignant tumor deaths also ranked first, accounting for 23.9% of the cases in China, in 2022 (2). The survival of lung cancer patients has significantly improved due to effective systems and advancements in multimodal therapy, encompassing surgery, radiotherapy, chemotherapy, immunotherapy, and molecular targeted therapy. Chemotherapy has emerged as a pivotal treatment modality for lung cancer patients (3). While it can alleviate clinical symptoms and extend survival, it may also induce various adverse reactions, including hair loss, bone marrow suppression, fatigue, nausea and vomiting, sleep disturbances, depression, and anxiety (4, 5). Nausea and vomiting represent the most prevalent and severe adverse reactions despite the existence of global and domestic guidelines for chemotherapy-induced nausea and vomiting (6, 7). These guidelines recommend combination medications for nausea and vomiting; however, the adherence to these guidelines in clinical practice remains suboptimal.

In a multicenter prospective cohort study about breast cancer patients, 44.9% and 12.8% of patients receiving highly emetogenic chemotherapy experienced nausea and vomiting in less than 24 hours afte administration, respectively, and more than 24 hours after administration, 59.4% and 44.6% of patients experienced nausea and vomiting (8).High emetogenic drugs are used in lung cancer patients during chemotherapy, they are still prone to nausea and vomitiing in accordance with the guidelines. It is a distressing concomitant symptom, which not only causes water and electrolyte disorders and malnutrition but also leads to the interruption of chemotherapy, thereby seriously affecting the patient’s quality of life and the implementation and efficacy of chemotherapy. Aerobic exercise and resistance and strength training can reduce nausea and vomiting symptoms during chemotherapy treatment of patients with tumors (9). Studies have shown that the severity and duration of nausea and vomiting improved and was significantly lower in patients enrolled in yoga programs while undergoing chemotherapy than those no in yoga programs (10, 11). However, the relationship between nausea and vomiting symptoms and physical activity during chemotherapy is unclear, and for patients with lung cancer using highly emetogenic chemotherapy drugs, the symptoms are severe during chemotherapy. Edbrooke et al. (12) revealed that the physical activity level decreased in patients with lung cancer undergoing chemotherapy, and Chemotherapy-related symptoms can significantly affect physical activity. However, its association with nausea and vomiting is unclear.

Therefore,we conduct a survey to study the status of physical activity and the incidence of nausea and vomiting after intravenous administration of chemotherapeutic agents. Moreover, we aimed to analyze the correlations between nausea and vomiting and physical activity in patients with lung cancer undergoing chemotherapy to provide evidence for follow-up intervention studies. And we can explore the right amount of exercise and improve the symptoms of chemotherapy-related nausea and vomiting in lung cancer patients, prevent or reduce the incidence of adverse reactions, prevent malnutrition, and finally, improve their quality of life.

## 2 Methods

### 2.1 Patient enrollment

Briefly, the parent study enrolled adults who were ≥18 years old with chemotherapy regimens containing platinum. Lung cancer were diagnosed by pathological analysis and initially received chemotherapy regimens containing platinum (cisplatin or carboplatin). All patients had understood the purpose of the study, volunteered to participate, and provided written informed consent. The exclusion criteria were as follows: patients undergoing hormone therapy those using prohibited drugs or alcohol, patients with gastric surgery and incomplete or complete intestinal obstruction, and those with nausea and vomiting not caused by chemotherapy. Therefore, 127 patients with lung cancer who received their first chemotherapy were enrolled in university hospitals and the cancer center in Shanghai. In addition, there were 20 cases with incomplete data, which were regarded as invalid samples. These patients refused to continue to answer the questionnaire, possibly because they were in a bad mood and felt uncomfortable during and after chemotherapy. Finally, only 107 patients with lung cancer were evaluated in the current analysis.

### 2.2 Data collection process

Individual interviews were conducted with lung cancer patients who hospitalized at A University Hospital where the study was conducted. Cross-sectional surveys and prospective clinical observations were used to investigate the patients who met the inclusion and exclusion criteria after obtaining informed consent (1). The general information questionnaire and the International Physical Activity Questionnaire (IPAQ) were completed following admission but before chemotherapy. This study aimed to evaluate the physical activity status of patients in the week before chemotherapy (2); Participants reported and INVR were recorded on the second and sixth day at 8pm after chemotherapy administration, and the average value of the two results was obtained to evaluate chemotherapy-induced nausea and vomiting (Literature review indicates that the symptoms of nausea and vomiting in patients with lung cancer were most obvious during the 2^th^–6^th^ day of the chemotherapy cycle (13); the second and sixth day after intravenous infusion of chemotherapy drugs represented acute and delayed nausea and vomiting, respectively. To clearly and concisely show the relationship between physical activity and nausea and vomiting symptoms, patients during the first chemotherapy were selected for this study).

### 2.3 Estimation by medical staff

The professionally trained medical staff had a face-to-face interaction with the patients, and the unified guidance language was used to explain the purpose and significance of the study and the relevant precautions in the confidentiality principles. The questionnaire was immediately collected after the patients filled it up, and it was checked and individually sorted. The questionnaire with missing items that could not be completed was considered invalid.

### 2.4 Instruments

#### 2.4.1 General information questionnaire

The questionnaire was designed by the researchers themselves, and it included characteristics such as age, gender, education level, marital status, smoking and drinking history, motion sickness history, and pregnancy-induced vomiting history. Additionally, disease-related data such as tumor staging, surgery, chemotherapy regimen, and antiemetic regimen were also included. On the day of patient enrollment, clinical data information was collected through face-to-face interviews and from the hospital’s electronic medical record system.

#### 2.4.2 Index of nausea, vomiting, and retching

The INVR scale (14) has eight items and three dimensions, including symptom experience time, symptom frequency, and symptom severity. Each dimension has different items and different scores for the three symptoms (nausea, vomiting, and retching). The scale adopted the 5-point Likert scale, with 0–4 points representing “not at all,” “some,” “moderate,” “very obvious,” and “very serious and unbearable.” The minimum and maximum cumulative scores were 0 and 32 points, respectively. The higher the score of each dimension, the more serious the degree of nausea, vomiting, and retching in patients.

INVR is a self-evaluation questionnaire and it is used for assessing the state of nausea,vomiting and retching in duration, frequency and severity (15). But there was no clear grading of the severity of patients’ overall self-perception,so it was graded according to the total score by Quan X W as follow (16).This classification was used to better account for the overall degree of nausea, vomiting and retching as follow.

No nausea, vomiting, or retching represented grade 0; ≤8 points represented grade I; 9–16 points represented grade II; 17–24 points represented grade III; >24 points represented grade IV. In 2002, experts from the Beijing Cancer Institute and the Chinese Academy of Medical Sciences translated INVR into Chinese, and they assessed the reliability and validity of the Chinese version of the RINVR scale. Cronbach’s α coefficient was 0.952, indicating good reliability and validity of the scale (17).

#### 2.4.3 Physical activity

IPAQ was used to measure the physical activity of the patients. The questionnaire included four dimensions: daily work, daily life, daily transportation, and exercise and leisure exercise. Medium to high levels of exercise entail various activities such as outdoor work-related tasks (paid work, volunteer work, farm activities, or studying), household chores (both indoor and outdoor), recreational pursuits, and daily commuting (whether by bicycle, motorcycle, train, or other means). Furthermore, separate assessments were made for sitting time during rest, including activities like reading, watching TV, socializing, and chatting. Walking time was accounted for within the aforementioned activities. Reliability and validity tests conducted across 12 countries confirmed the soundness of the two questionnaires (18). The Chinese version underwent revisions and was primarily used to gauge the frequency and duration of physical activities of different intensities over a 7-day period by Ningning Qu et al. (19). Mengyu Fan et al. (20) assigned MET values to physical activities and proposed a calculation method where an individual’s weekly level of physical activity of a certain intensity is determined by multiplying the corresponding MET value by the weekly frequency (days per week) and the daily duration (minutes per day). Based on weekly energy expenditure, the questionnaire categorizes physical activity levels into three categories: low, medium, and high (1). High physical activity group: high-intensity occupational or recreational activity on ≥3 d and achieving 1500 MET-min/week or a combination of various activities for ≥7 d (2); Medium physical activity group: high-intensity occupational or recreational activity on ≥3 d with 2 types of activity for ≥60 min each or medium-intensity occupational or recreational activity and traffic activity for ≥7 d with 3 types of activity for 150 min or a combination of various activities on ≥5 d, achieving a MET ≥600 (3); Low physical activity group: any physical activity MET value that failed to meet the inclusion criteria of high- and medium-intensity levels. The questionnaire is one of the internationally recognized, effective, and widely used physical activity level measurement questionnaires and commonly used to measure the physical activity level of patients with lung cancer (21).

### 2.5 Sample size calculation and statistical analysis

According to the preliminary test results, the occurrence of dry vomiting and nausea and vomiting symptoms in patients with lung cancer undergoing chemotherapy was approximately 50%. According to the formula N = Z^2^_α_P(1−P)/d^2^ for sample size calculation, the α was set to 0.05, and the power value was set to 80%. The PASS15.0 software was used to calculate the required sample size of 97 cases. Considering the dropout rate of approximately 10%, the sample size was determined to be 107 cases.

The SPSS 22.0 was used for statistical analysis. Categorical variables were represented using frequency and rate (%). The difference between groups was compared using the χ^2^-test. The distribution of the degree of nausea and vomiting was conducted using the χ^2^- test, and the correlation between the degree of nausea and vomiting and physical activity was analyzed using the multivariate unconditional logistic regression analysis. Statistical significance was set at P <0.05.

## 3 Results

### 3.1 General information on patients with lung cancer

A total of 107 patients with lung cancer were investigated in this study. Of these, 60 (56.07%) were men, and higher than women; the age range was 51–75 (average, 52.85 ± 6.98) years; 87 (81.31%) were urban patients, and 20 (18.69%) were rural patients; 56 (52.22%) had a history of smoking, and 104 (97.19%) were married;

A healthy weight range means a normal range of body mass index (BMI). Only two patients had a body mass index more than 24kg/m^2^. The proportion of patients with a BMI more than 18.5 and less than 23.9 was 98.13%.

The proportion of patients with normal body mass index was 100%; the education level was the lowest in 5 cases, accounting for 4.67%; 104 (97.19%) and 3 (3.81%) patients were nonworking and working, respectively; 38 (35.51%) possessed chronic diseases and 95 (88.78%) had previously undergone pulmonary surgery; the clinical stage was mainly concentrated in stage III and IV, with 56 and 44 cases accounting for 52.33% and 41.12%, respectively, and 7 cases of stage II patients accounted for 6.54%; all patients were treated with conventional antiemetic therapy before chemotherapy (metoclopramide + ondansetron + dexamethasone).

### 3.2 Distribution of nausea and vomiting in patients with lung cancer after chemotherapy

Among the patients having lung cancer treated with platinum-based chemotherapy, 49 were LeveI of INVR I, including 45 with a score of 0, 44 cases in LeveII of INVR, 11 cases in LeveIII of INVR, and 3 cases in LeveIV of INVR. The proportion of lung patients with no symptoms of nausea, vomiting and retching was only 42.05%. More than half of the patients experienced nausea, vomiting or retching related symptoms after chemotherapy.

**Figure 1:**
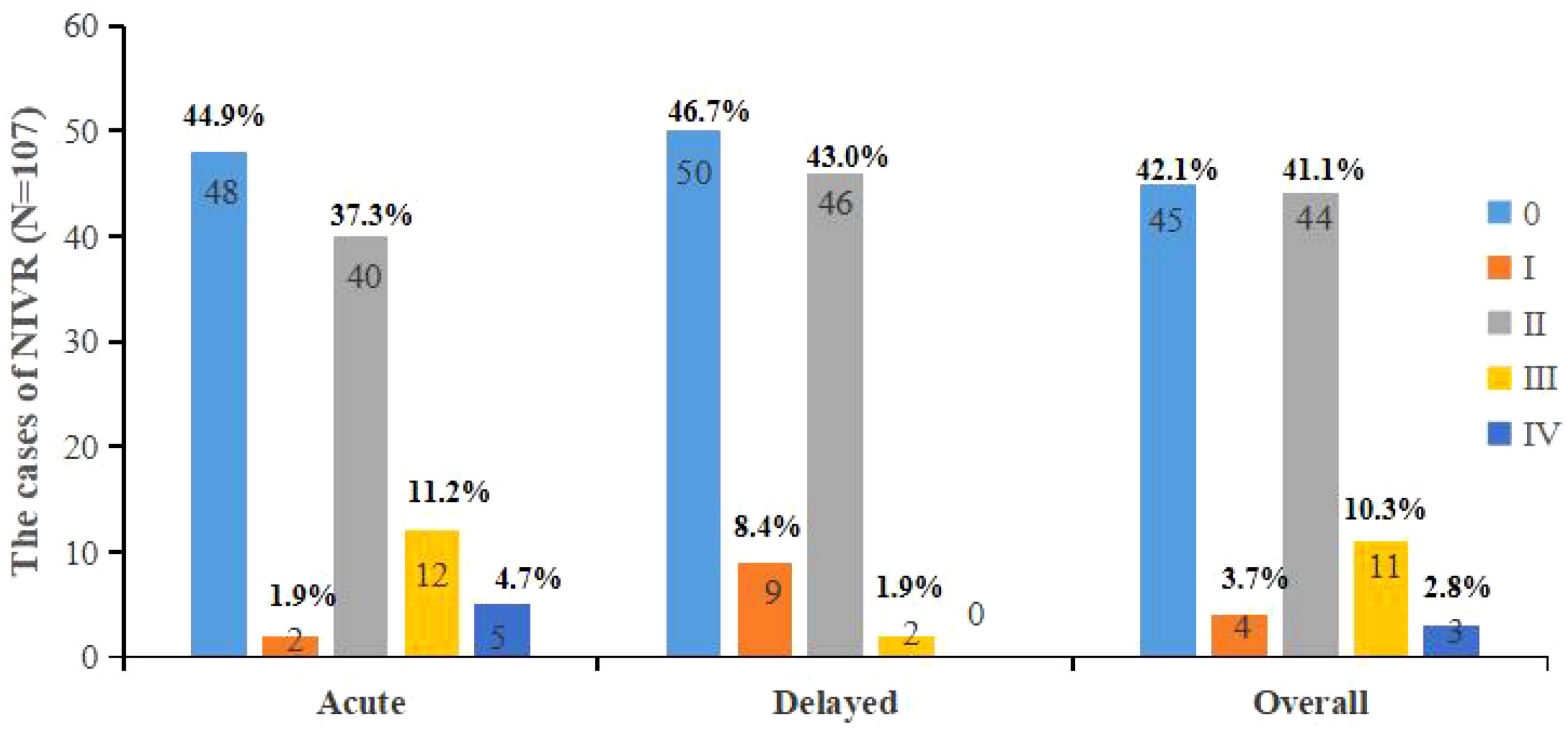
Incidence of INVR after chemotherapy.

The level of nausea and vomiting on day 2 after chemotherapy was as follows: 50 were LeveI of INVR I, including 48 with a score of 0, 40 cases in LeveII of INVR, 12 cases in LeveIII of INVR, and 5 cases in LeveIV of INVR; And the level of nausea and vomiting on day 6 after chemotherapy was as follows: 59 were LeveI of INVR I, including 50 with a score of 0, 46 cases in LeveII of INVR, 2 cases in LeveIII of INVR, and no in Leve IV of INVR (**Figure 1**).

Univariate analysis showed that age, gender, and history of drinking were the influencing factors for the degree of nausea and vomiting (P < 0.05).

### 3.3 Comparison of the degree of nausea and vomiting in patients having lung cancer with different physical activities

**Figure 2:**
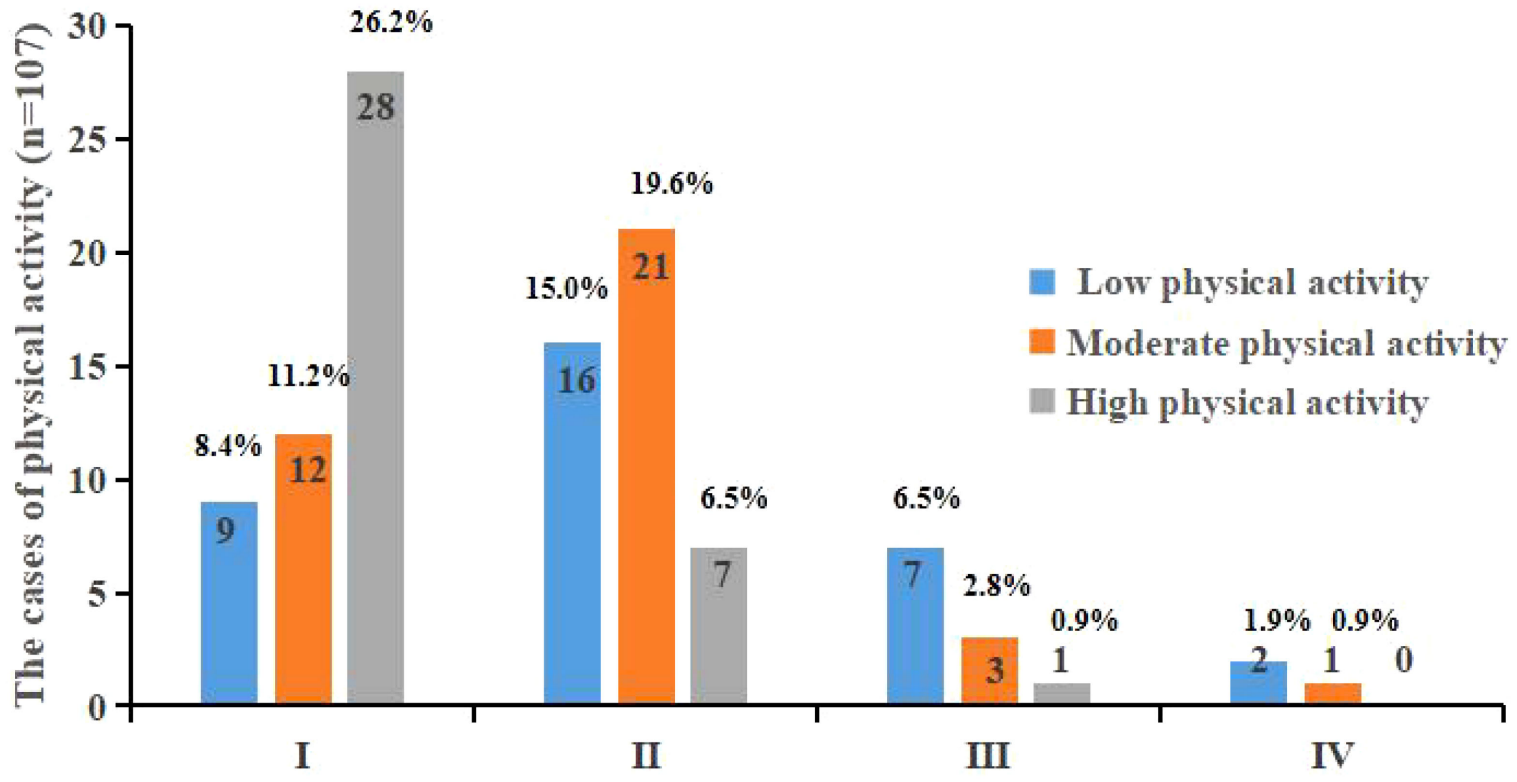
Physical activity in different INVR patients after chemotherapy.

There were 53 (49.5%), 37 (34.6%), and 17 (15.9%) patients with low, moderate, and high physical activity, and 50.5% had moderate to high physical activity accounts for the majority of nausea and vomiting in grade I and II (**Figure 2**). Statistically significant differences were observed between patients having lung cancer with different physical activities and the degree of chemotherapy-related nausea and vomiting (P<0.05).

### 3.4 Multivariate linear analysis of the degree of nausea and vomiting and physical activity in patients with lung cancer

The results of univariate analysis show that gender, age, drinking history, and the level of physical activity are the influencing factors of nausea and vomiting (P < 0.05). Dummy variables were set for multi-categorical variables, and the way of assignment was shown (**Table 1.1**). The mean INVR was used as the dependent variable. The variables with statistically significant differences in univariate analysis were considered as independent variables, with α in =0.05 and α out =0.10, and multivariate linear regression analysis was performed. The regression equation was established and tested, F=14.995, P<0.001, indicating that the regression equation was statistically significant.

**Table 1.1:** The assignment of each variable in multivariate linear regression analysis.

variable	Details	Assign value
Y	INVR	Total Score
X1	Gender	Man=1, Female=2
X2	Age	<30 = 1,30–50 = 2,51–70 = 2,>70 = 3
X3	History of drinking	Yes=1,No=2
X4	Moderate physical activity	Total Score
X5	High physical activity	Total Score

The adjusted R2 of the coefficient of determination was 0.369, indicating that the regression model composed of gender, drinking history, moderate physical activity, high physical activity and nausea and vomiting could explain 36.9% of the total variation of nausea and vomiting in lung cancer patients undergoing chemotherapy.

**Figure 3:**
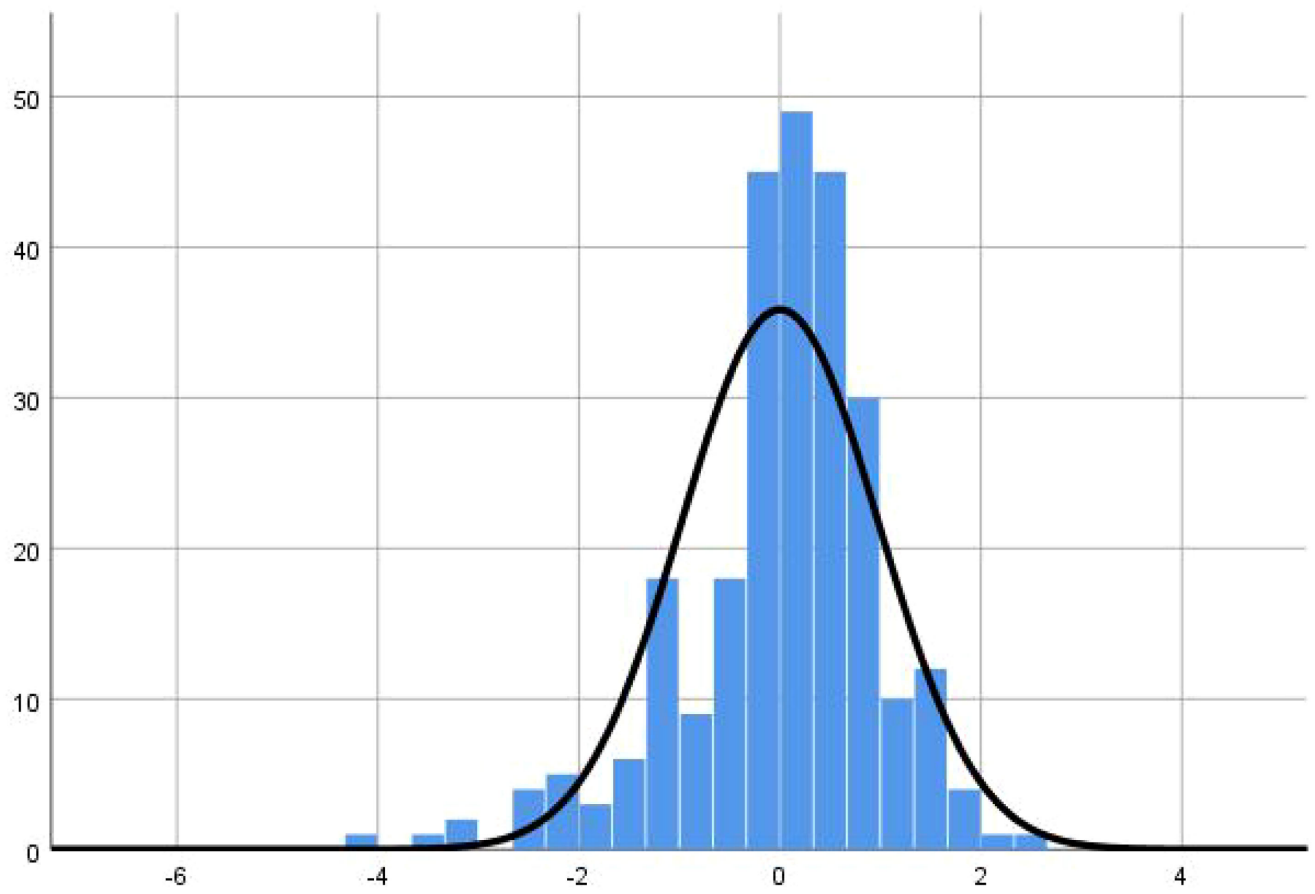
Standardized residual of the INVR.

According to multivariate linear regression analysis, the protective factors associated with chemotherapy-related nausea and vomiting in patients with lung cancer were gender, drinking history, moderate physical activity, and high physical activity (P < 0.05) (**Table 1.2**).Multivariate linea analysis of the residual figure of nausea and vomiting is the standardized residual graph (**Figure 3**).

**Table 1.2:** Multivariate linear analysis of the degree of nausea and vomiting and physical activity(n=107).

Model	β	SE	Beta	t-*Value*	P
Constant	7.937	1.161	-	6.839	0.000
Gender	0.347	0.124	0.145	2.808	0.005
Age	0.166	0.115	0.075	1.440	0.151
History of drinking	-1.907	0.958	-0.210	-2.011	0.021
Moderate physical activity	0.250	0.035	0.458	7.096	0.000
High physical activity	0.029	0.012	0.195	2.525	0.012

SE, standard error.

## 4 Discussion

Nausea and vomiting are a common adverse reaction during chemotherapy, which is the most feared reaction in patients with lung cancer during chemotherapy. Nausea and vomiting can result in severe side effects and reluctance to continue treatment. Despite the use of antiemetic drugs for prevention prior to chemotherapy, effective prevention and management of nausea and vomiting still fall short of meeting clinical needs. This study revealed that the incidence of nausea, vomiting, and retching during chemotherapy was 57.94% (**Table 2**), consistent with findings from previous research published elsewhere (22). Mou Qianqian et al. demonstrated that following the administration of conventional antiemetic drugs, the incidence of acute nausea and vomiting induced by initial platinum chemotherapy in lung cancer patients was 42.7%, with a delayed incidence of nausea and vomiting reaching 61.6% (23).

**Table 2:** Distribution of patients’ general characteristics among nausea and vomiting groups [n (%)].

Variable	Leve I	Leve II	Leve III	Leve IV	χ2	P
Gender						
man	39(54.2)	31(43.1)	1(1.4)	1(1.4)	22.068	0
women	10(28.6)	13(37.1)	10(28.6)	2(5.7)		
age, years						
<30	0(0.0)	1(50.0)	1(50.0)	0(0.0)	26.522	0.002
30–50	8(20.5)	27(69.2)	3(7.7)	1(2.6)		
51–70	34(61.8)	12(21.8)	7(12.7)	2(3.6)		
>70	7(58.3)	5(41.7)	0(0.0)	0(0.0)		
Livingstyle						
city	25(45.5)	23(41.8)	5(9.1)	2(3.6)	0.452	0.929
Village	24(46.2)	21(40.4)	6(11.5)	1(1.9)		
History of smoking						
Yes	12(33.3)	22(61.1)	1(2.8)	1(2.8)	6.896	0.075
No	18(36.0)	20(40.0)	10(20.0)	2(4.0)		
History of drinking						
Yes	41(53.2)	30(39.0)	5(6.5)	1(1.3)	9.692	0.021
No	8(26.7)	14(46.7)	6(20.0)	2(6.7)		
Marital status						
married	39(42.9)	40(44.0)	9(9.9)	3(3.3)	3.946	0.684
divorce	2(50.0)	1(25.0)	1(25.0)	0(0.0)		
Widowed spouse	8(66.7)	3(25.0)	1(8.3)	0(9.0)		
Educational						
Primary school	13(43.3)	12(40.0)	4(13.3)	1(3.3)	10.371	0.321
Junior high school	11(30.6)	18(50.0)	6(16.7)	1(2.8)		
Senior high school	17(48.6)	8(51.4)	0(0.0)	0(0.0)		
College or above	8(50.0)	6(37.5)	1(9.4)	1(6.3)		
Historyofchronicdiseases						
Yes	30(45.5)	28(42.4)	6(9.1)	2(3.0)	0.344	0.952
No	19(46.3)	16(39.0)	5(12.2)	1(2.4)		
Operation						
Yes	23(38.3)	27(45.0)	8(13.3)	2(3.3)	3.535	0.316
No	26(55.3)	17(36.2)	3(6.4)	1(2.1)		
Tumor stage						
Stage I	4(66.7)	2(33.3)	0(0.0)	0(0.0)	7.191	0.617
Stage II	12(54.5)	7(31.8)	3(13.6)	0(0.0)		
Stage III	18(39.1)	19(41.3)	7(15.2)	2(4.3)		
Stage IV	15(45.5)	16(48.5)	1(3.0)	1(3.0)		

When lung cancer patients received three antiemetics during chemotherapy, the incidence of nausea was high, with the incidence of nausea and vomiting more than 50% and 20% in the overall phases of chemotherapy, respectively (13, 24).Other patients receiving highly emetogenic drugs experienced a notably high incidence of delayed nausea and vomiting, with the severity of nausea being most pronounced on the first day post-chemotherapy (25). This study revealed that nausea and vomiting incidence peaked on the second day after chemotherapy, with level IV incidence at 2.80% and level I at 45.79% (**Table 2**). However, anti-tumor drugs administered to lung cancer patients pose a high risk of vomiting, which proves challenging to control and persists for an extended duration. Therefore, patients required intravenous supportive care for nausea and vomiting during chemotherapy, leading to a rise in overall monthly economic and medical costs (26). The symptoms of vomiting were not only causing water-electrolyte disturbances and malnutrition but also significantly harming the emotional and social well-being. In addition, it affected the treatment effect and survival time (27). Thus, greater attention should be paid to patients with lung cancer undergoing chemotherapy, despite the routine use of antiemetic drugs before chemotherapy. Nausea and retching were still very painful.They can deterioration of patient quality of life.

Chemotherapy is a common cancer treatment, often accompanied with side effects, such as nausea and vomiting. Nausea and vomiting in patients with lung cancer undergoing chemotherapy are not only related to age, gender, history of drinking, chemotherapy regimen, and antiemetic regimen but also related to the psychological status, nutritional status, and sleep of the patients (28). Physical fitness levels have been identified as independent predictors of survival. Additionally, physical activity has been demonstrated to alleviate fatigue, enhance quality of life, improve pulmonary function, increase muscle mass and strength in lung cancer patients (29). Regular physical activity appears to be correlated with improved mood and reduced symptom burden in these patients (30). Considering physical activity as a key patient-reported outcome, its impact on nausea and vomiting symptoms during chemotherapy warrants attention. Research indicates that the weekly level of physical activity influences the potential categories of chemotherapy-related symptom clusters in lung cancer patients, leading to improvements in lung muscle strength, reduced dyspnea, fatigue, pain, and discomfort (31, 32). The symptom cluster includes loss of appetite, taste changes, nausea, and vomiting, observed across chemotherapy cycles and within a single cycle (33).

This study found that the primary mode of activity among lung cancer patients undergoing chemotherapy was walking, with half of patients engaging in low physical activity in the past week (**Table 3**). This trend may be associated with the older age of the patients. The severity of the symptoms related to chemotherapy was inversely proportional to the physical activity level of patients with lung cancer, and patients having more severe symptoms benefited the most from physical activity (34), thereby showing that physical activity is beneficial to patients with lung cancer. Sloanga et al (35) showed that patients with lung cancer undergoing chemotherapy in the early stage of the disease and with fewer complications are more likely to engage in physical activity compared with those with serious nausea and vomiting symptoms who had lower physical activity levels. The present study showed that the incidence of nausea and vomiting in the low-level group was significantly higher than that in the moderateand high-level groups (P < 0.05) (**Table 3**), showing that more than moderate-level physical activity is required to obtain a certain effect on improving nausea and vomiting symptoms in patients with lung cancer undergoing chemotherapy. The results of the univariate analysis showed that the daily activity time of moderate to high-intensity physical activity was related to the degree of nausea and vomiting (P<0.05). Moreover, women demonstrated more severe nausea and vomiting than men; moderate to high physical activity was associated with lower degree of nausea and vomiting. The potential reasons for the association between physical activity and nausea and vomiting symptoms are as follows: Physical activity can modulate the excitatory and inhibitory functions of the cerebral cortex, thereby regulating the patient’s mood. Through neuro-endocrine regulation, the release of brain neurotransmitters and their receptors induces physical and mental relaxation in the body, while promoting the release of endorphins, reducing various physical discomforts, and eliciting a sense of pleasure. Multivariate analysis results indicated that drinking history, gender, and patients’ daily activity levels were influencing factors of nausea and vomiting (**Table 2**). There exists a bidirectional relationship between physical activity and nausea and vomiting symptoms, with physical activity significantly promoting these symptoms. However, intense exercise may exacerbate gastrointestinal reactions, such as nausea and vomiting (36). Medium to high-intensity aerobic exercise and resistance training can mitigate fatigue and other adverse reactions experienced by patients during chemotherapy, facilitating their rehabilitation (37). Simple physical activity, such as walking or cycling, is relatively easy to accomplish, which not only reduces nausea and vomiting but also improves the quality of life of patients with lung cancer.

**Table 3:** Comparison of nausea and vomiting in patients with different levels of physical activity [n (%)].

Variable	Leve I	Leve II	Leve III	Leve IV	χ2	P
Low physical activity	28 (52.8)	16 (30.2)	7 (13.2)	2 (3.8)	45.257	0.005
Moderate physical activity	12 (32.4)	21 (56.8)	3 (8.1)	1 (2.7)		
High physical activity	9 (52.9)	7 (41.2)	1 (5.9)	0 (0.0)		

This study has some limitations. Firstly, the sample size is small. The research participants are patients who used platinum therapy for the first time, with the research time being only one chemotherapy cycle. Secondly, some factors, such as anxiety, depression, and insomnia, may affect physical activity. The lack of these factors in this study may affect the degree of chemotherapy-induced nausea and vomiting.Future work is required to collect relevant information and to explore the effects on physical activity and nausea and vomiting.In addition, the cases of multiple chemotherapy cycles could be further investigated.

## 5 Conclusion

The incidence of nausea and vomiting in lung cancer patients during chemotherapy was higher, and the proportion of moderate to high physical activity before chemotherapy is a protective factor for nausea and vomiting in patients with lung cancer, thereby reducing the degree and incidence of nausea and vomiting in patients with lung cancer during the first chemotherapy. Long-term moderate to high physical activity is beneficial to reduce nausea and vomiting during the first chemotherapy.

## Data availability statement

The original contributions presented in the study are included in the article/supplementary material. Further inquiries can be directed to the corresponding authors.

## Ethics statement

The studies involving humans were approved by the Ethics Committee of East Hospital Affiliated to Tongji University, batch number (2023) No. 001. The studies were conducted in accordance with the local legislation and institutional requirements. Written informed consent for participation was not required from the participants or the participants’ legal guardians/next of kin in accordance with the national legislation and institutional requirements. Written informed consent was obtained from the individual(s) for the publication of any potentially identifiable images or data included in this article.

## Author contributions

LB: Writing – original draft, Writing – review & editing, Methodology, Project administration, Resources. XD: Supervision, Project administration, Writing – review & editing. WZ: Data curation, Writing – review & editing. JL: Investigation, Software, Resources, Writing – review & editing. NL: Writing – review & editing, Formal analysis, Investigation. YZ: Writing – review & editing. YY: Data curation, Writing – original draft.
